# Lipopolysaccharide core type diversity in the *Escherichia coli* species in association with phylogeny, virulence gene repertoire and distribution of type VI secretion systems

**DOI:** 10.1099/mgen.0.000652

**Published:** 2021-09-29

**Authors:** Sébastien O. Leclercq, Maxime Branger, David G. E. Smith, Pierre Germon

**Affiliations:** ^1^​ UMR ISP, INRAE, Université François Rabelais de Tours, F-37380 Nouzilly, France; ^2^​ Institute for Biological Chemistry, Biophysics and Bioengineering (IB3), Riccarton Campus, Heriot-Watt University, Edinburgh EH14 4AS, UK

**Keywords:** LEE, LPS core types, phylogeny, STc131, type VI secretion system, virulence

## Abstract

*
Escherichia coli* is a very versatile species for which diversity has been explored from various perspectives highlighting, for example, phylogenetic groupings and pathovars, as well as a wide range of O serotypes. The highly variable O-antigen, the most external part of the lipopolysaccharide (LPS) component of the outer membrane of *E. coli *, is linked to the innermost lipid A through the core region of LPS of which five different structures, denominated K-12, R1, R2, R3 and R4, have been characterized so far. The aim of the present study was to analyse the prevalence of these LPS core types in the *E. coli* species and explore their distribution in the different *E. coli* phylogenetic groups and in relationship with the virulence gene repertoire. Results indicated an uneven distribution of core types between the different phylogroups, with phylogroup A strains being the most diverse in terms of LPS core types, while phylogroups B1, D and E strains were dominated by the R3 type, and phylogroups B2 and C strains were dominated by the R1 type. Strains carrying the LEE virulence operon were mostly of the R3 type whatever the phylogroup while, within phylogroup B2, strains carrying a K-12 core all belonged to the complex STc131, one of the major clones of extraintestinal pathogenic *E. coli* (ExPEC) strains. The origin of this uneven distribution is discussed but remains to be fully explained, as well as the consequences of carrying a specific core type on the wider aspects of bacterial phenotype.

## Data Summary

All supplementary material has been deposited at FigShare: https://doi.org/10.6084/m9.figshare.14773392.v1. A complete list of strains with associated data is available in Table S1 (also available with the online version of this article). A list of virulence genes tested and a complete table for the presence/absence of virulence genes are available in Tables S2 and S3. A complete list of virulence genes in each virulence gene group is presented in Table S4.

**Impact Statement**

The diversity of the *Escherichia coli* species in terms of type of infections is well known, and its surface O-antigen, which shows some correspondence to pathogenicity. This study is believed to be the first one to report in detail the diversity and distribution of the lipopolysaccharide (LPS) core types in the *E. coli* species using whole-genome sequences. This distribution of the five know core types is analysed in the different phylogroups along with their association with specific virulence gene repertoires. Results indicate a non-random distribution of LPS core types in the *E. coli s*pecies and, most interestingly, preferential association of certain core types with either some phylogenetic clades or particular virulence gene repertoires. This analysis highlights a complexity in the relationship between LPS core types, O-antigen types, phylogeny and selected pathotype determinants, and indicates that further examination of interactions among these characteristics is required to establish the influence on bacterial phenotypes.

## Introduction

The species *Escherichia coli* is characterized by the great diversity of the strains it encompasses. This diversity is illustrated, phenotypically, by more than 180 different serogroups of O-antigen described, as well as by the diversity of commensals and pathovars [1–3]. Indeed, phenotypic differences translate in different capacities to cause, or not, infections. Most *E. coli* strains are considered as commensals of warm-blooded animals, with an additional reservoir in the environment [3, 4]. Still, pathogenic *E. coli* isolates have long been described in different hosts and are responsible for a variety of intestinal and extra-intestinal infections [1, 5, 6].

Phylogenetically, *E. coli* strains have been classified in seven major phylogroups termed A, B1, B2, C, D, E and F, with an eighth recently identified as phylogroup G [1, 3, 7]. Although not absolute, some preferential associations exist between certain phylogenetic groups and pathovars. As an example, extraintestinal pathogenic *E. coli* (ExPEC) strains belong more frequently to the B2 and D phylogroups, while enterohaemorrhagic *E. coli* (EHEC) strains most often belong to the E group, but also belong to B1 and A groups [8].

A characteristic that has been so far overlooked in the study of *E. coli* diversity is that of lipopolysaccharide (LPS) core types. LPS is the major component of the outer leaflet of the outer membrane of *E. coli* [9]. The innermost part of LPS is the lipid A, to which is attached the core region of LPS of which five different structures have been characterized so far and, at the most external part, the highly variable O-antigen made of repeated polysaccharide subunits.

LPS core types are encoded by the *waa* locus genes located approximately 100 kb from the origin of replication in the *E. coli* species. The five distinct regions share highly similar 5′ and 3′ regions, encompassing *waaDFC* and *waaA-waaQGP* genes, while showing variable regions in between with lower G+C mol% values (Fig. S1) [10].

Reasons to investigate the diversity of LPS core types stem in part from the multiple properties conferred by LPS to the bacteria. The LPS is responsible for the weak permeability of the outer membrane (OM), preventing the entry of a number of toxic compounds such as antibiotics and, thus, acts as a protective barrier for bacteria [11]. O-antigen is a key determinant in the resistance of bacteria to complement mediated lysis [12, 13].

Being a conserved structure of Gram-negative bacteria, the lipid A part of LPS is one of the major bacterial constituents efficiently recognized by the innate immune system of the host [14]. This recognition is mediated by interaction with different host receptors, in particular the TLR4 receptor, and is modulated by the presence of the O-antigen. The core part of LPS has also been shown to regulate the interaction with host cells through the DC-SIGN receptor [15, 16]. Nevertheless, this recognition seems to be restricted to the K-12 LPS core type and linked to the presence of specific *N*-acetylglucosamine epitopes [15, 16]. Although this has not been studied in detail, one could speculate that other host interaction properties might depend on the type of core LPS.

So far, the distribution of LPS core types has been investigated in a restricted number of reports. The R1 type is the most prevalent of all core types, with R2, R3, R4 and K-12 core types having significantly lower prevalences [17–19].

Using a set of mAbs, Gibb and colleagues investigated the distribution of LPS core types in *E. coli* strains from diverse origin. Most strains were R1 type, with a higher percentage of R1 type in isolates from urine, compared to isolates from faeces and blood cultures [20]. Conversely, Dissanayake *et al.* found no difference in the distribution of LPS core types in commensal and pathogenic strains [18]. A potential link between virulence properties and LPS core types is also suggested by the observation that O157:H7 and verotoxin-producing *Escherichia coli* (VTEC) strains are in general of the R3 type [21–23]. These reports also showed an uneven distribution of LPS core types between phylogroups. While phylogroup B2 and D strains, which encompass a range of ExPECs, were found restricted to R1 types, all four other core types were represented among phylogroup A strains, still with a majority of strains being R1.

Hence, we wished to establish, based on genome sequence analysis, the prevalence of the different LPS core types in the *E. coli* species and, simultaneously, analyse the potential relationships between LPS core types, phylogenetic groups and the virulence genes repertoire.

## Methods

### Choice of strains

A set of 500 genome sequences from 499 *E. coli* strains and 1 *Escherichia albertii* were downloaded from GenBank on 14th October 2018. The *E. coli* genome sequences were randomly chosen among the 13 250 sequences available in GenBank for this species when his study was initiated.

### Phylogenetic analysis

A maximum-likelihood (ML) phylogenetic tree covering the different strains of our study was obtained by analysis of the pan-genome SNPs (1157369 positions) using the kSNP3 (v3.92) program [24], itself calling FastTree2 (v2.1.3) [25]. Results from this initial analysis were assessed to identify groups of clonal strains whose genome sequences were different by less than 10 SNPs among the 7008 SNP positions identified in all 499 strains. Only one strain per group was kept, which led to a set of 269 *E. coli* strains. A new pan-genome SNP analysis (974791 polymorphic positions) was performed using the kSNP3 program to produce a ML phylogenetic tree for these non-clonal strains. The strain of *E. albertii* was included as an outgroup. Graphical representation of the ML tree was performed using the iTOL web server [26].

### *In silico* LPS core type determination, ECOR and serogroup typing

The type of core LPS was analysed based on the presence of different alleles of the *waaL* gene, as described previously [27]. *In silico* ECOR typing was performed using the ClermonTyping script [28]. Serogroup analysis was performed using serotype-specific sequences from ECTyper (https://github.com/phac-nml/ecoli_serotyping). The phylogroup determined by the ClermonTyping was, for a few strains, not consistent with the phylogenetic clustering performed by kSNP. In such situations, the kSNP clustering was retained. Sequence type (ST) and ST complex (STc) analyses were performed using the MLSTar package (v 0.1.3) using the Achtman scheme, and STc was allocated based on the PubMLST database (https://pubmlst.org).

### Virulence gene repertoire analysis

*E. coli* virulence protein sequences (Table S2) were collected from the ecoli_VF_collection (https://github.com/aleimba/ecoli_VF_collection) [29] and a presence/absence matrix (Table S3) was generated as described by Kempf *et al.* [27]. Briefly, a perl script was used to run tblastn (v2.10.1) on each individual genome for the set of virulence genes tested. A gene in a strain was considered present when the best hit showed at least 80 % similarity and 80 % coverage, as described in other studies [30]. This resulted in a large matrix, with one line per strain and one column per virulence gene, in which presence was encoded as 1 and absence as 0. Clustering of strains according to virulence gene repertoire was performed using Rstudio (v1.3.1056), R (4.0.2) and the R package pheatmap (v1.0.12). Prior to clustering genes based on their presence/absence profiles, because they are likely not to contribute to the clustering analysis as they are present or absent in most strains without distinction of phylogroup, we removed genes that were present in more than 90 % of strains of each phylogroup as well as genes present in less than 30 % of strains of each phylogroup. From the 1069 genes in the initial panel, only 579 remained after this filtering step.

The optimal number of clusters was determined using the eclust R package (v0.1.0). Distance used in clustering analyses was binary (1 for presence and 0 for absence of the corresponding gene) and the method of aggregation was Ward.D2. Thus, the optimal number of clusters was determined to be 43. For each strain, the enrichment score of each group of genes was calculated by counting the number of genes from each group present in this strain. Principal component analysis (PCA) was performed based on the enrichment score of each group of genes using the FactoMiner R package (v2.3).

### Association analyses

For each type of core, a 2×2 contingency table was generated with the number of strains possessing, or not, the analysed specific core and the number of strains positive for each virulence gene group (VGG). A strain was considered positive for a VGG when its enrichment score was higher than 0.7. The *P* value for the probability that each contingency table was a random distribution was calculated by a Fisher's exact test using the stats (v4.0.2) R package. *P* values were then adjusted for multiple comparisons by the Bonferroni method. Heatmaps of *P* values were generated using the pheatmap package (v1.0.12).

## Results

### LPS core types are not evenly distributed in the different phylogroups

The aim of this study was to investigate the relationship between the different types of LPS core, the phylogenetic group and the carriage of virulence genes in the *E. coli* species. To this end, we first randomly selected 500 *Escherichia* genomes in GenBank including 499 *E. coli* genomes and 1 *E. albertii* genome. Despite being randomly selected, an initial SNP analysis suggested that some strains might be clonal. Because of this potential clonality, before undertaking more in-depth studies, we removed from our dataset strains that could be considered as representative of the same clones and only one strain per such group was kept (see Methods). This filtering resulted in a set of 269 strains to which was added the *E. albertii* genome as an outgroup.

**Fig. 1.**
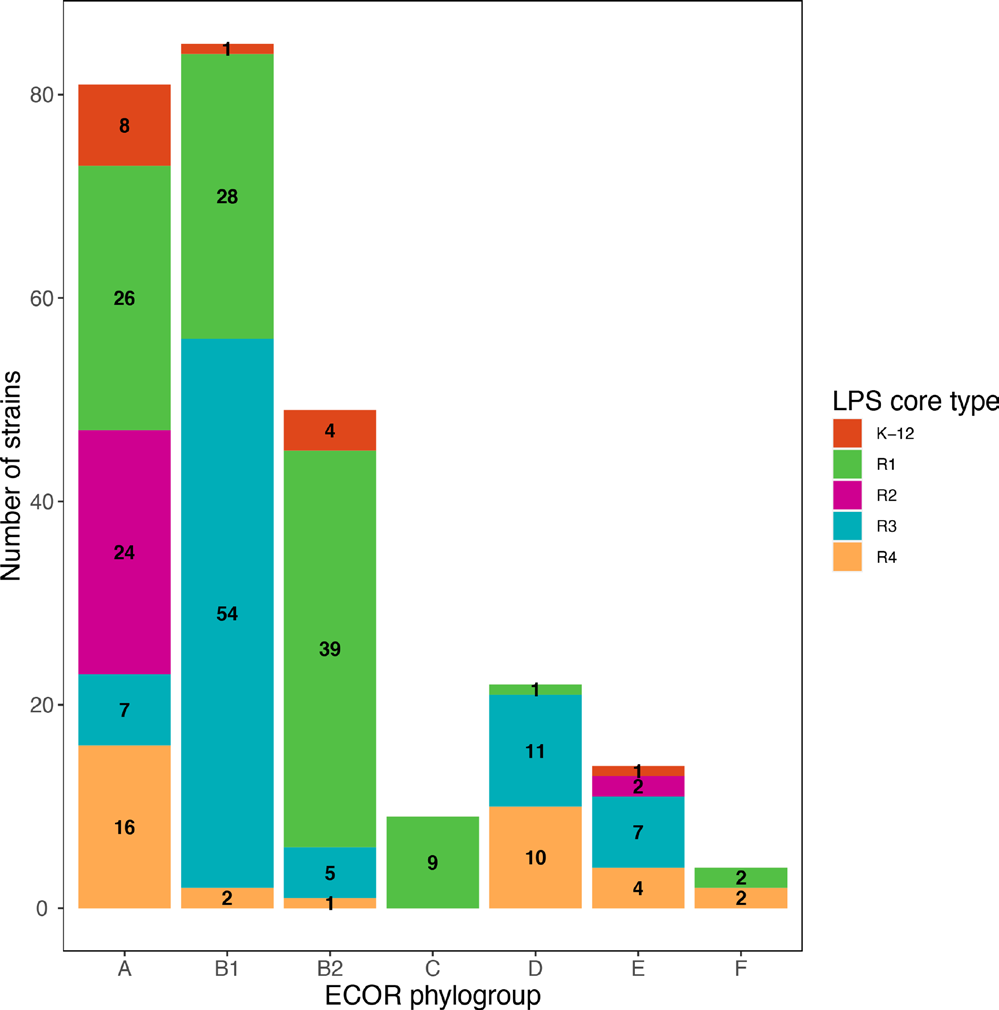
Prevalence of the different LPS core types in *E. coli* strains. Core LPS was determined by sequence analysis of core-specific *waaL* alleles. Core type could not be determined for five strains.

Distribution of strains among the main *E. coli* phylogroups in this dataset of 269 strains was as follows: 83 phylogroup A strains (30.7 %), 87 B1 strains (32.2%), 49 B2 strains (18.1 %), 10 C strains (3.7 %), 22 D strains (8.1 %), 14 E strains (5.2 %), 4 F strains (1.5 %) (Fig. 1). Prevalence of the different LPS core types in this set of *E. coli* strains was 14 (5.2 %) for K-12, 105 (38.9 %) for R1, 26 (10.0 %) for R2, 84 (31.1 %) for R3, 35 (13.0 %) for R4. LPS core type could not be attributed for five strains (1.9 %).

**Fig. 2.**
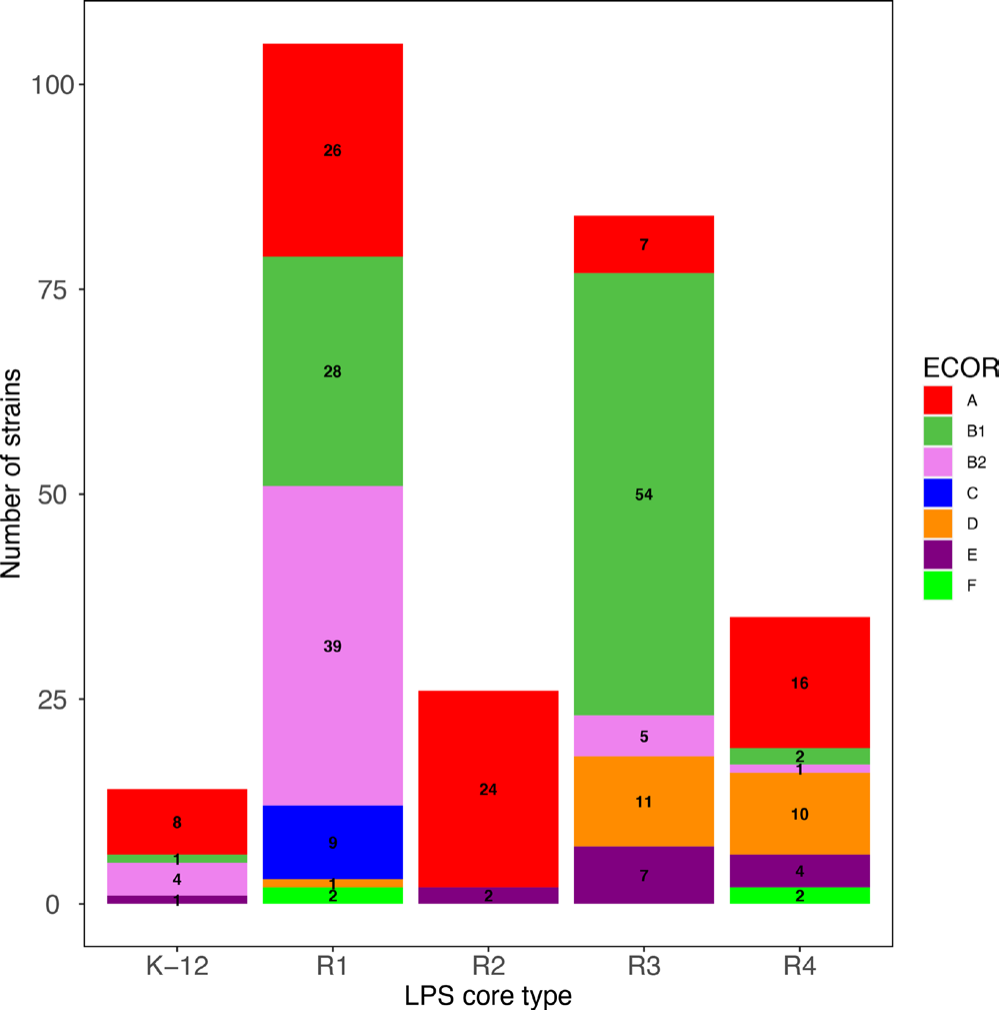
Distribution of LPS core types in major *E. coli* phylogroups. ECOR phylogroup was determined based on kSNP analysis of genome sequences and *in silico* ECOR typing. Core LPS was determined by sequence analysis of core-specific *waaL* alleles. Only strains belonging to *E. coli* phylogroups A, B1, B2, C, D, E and F are shown.

The prevalence of LPS core types in the different *E. coli* phylogroups was then analysed in two ways (Figs 1 and 2), both of which showed that the proportion of the different LPS core types was highly variable depending on the phylogroup of the strains. This distribution is non-random as indicated by a Fisher's exact test (*P*<0.001). Phylogroup A strains are very diverse in terms of LPS core types and have somewhat similar proportions of all five LPS core types. R2 strains were mostly found in phylogroup A strains, with two exceptions found in phylogroup E. Strains belonging to the other phylogroups are less diverse with, for example, more strains of the R3 core types in phylogroups B1, D and E. A majority of strains of the B2 phylogroup, as well as all strains of phylogroup C, were of the R1 LPS core type. Finally, the few F strains of our set were either of the R1 or R4 core type.

**Fig. 3.**
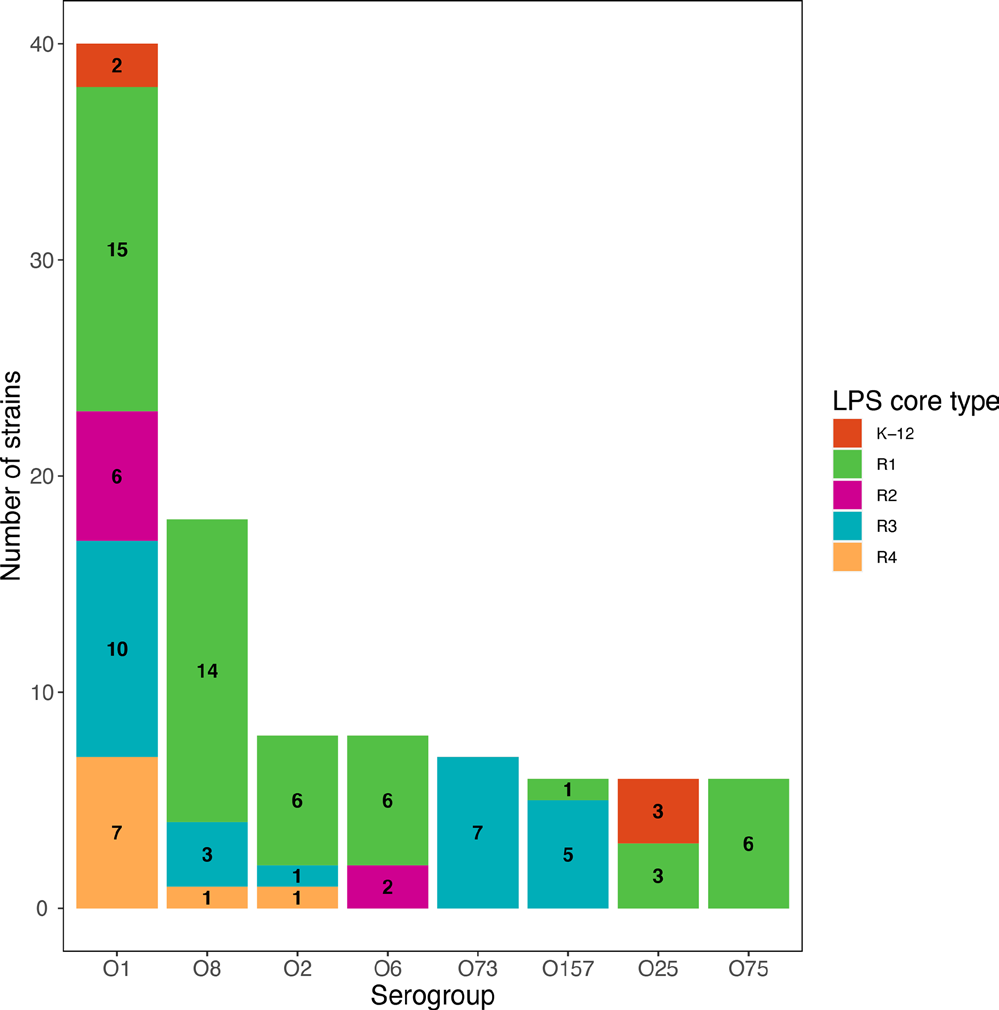
Distribution of LPS core types depending on strain O serogroup. O serogroup was determined based on sequence analyses for O-serogroup-specific sequences. Core LPS was determined by sequence analysis of core-specific *waaL* alleles. Only serogroups with more than 10 strains are represented.

The serogroup of strains was also determined *in silico*. We observed that some serogroups were preferentially associated with certain LPS core types (Fig. 3). For example, strains of serogroup O157 and O73 were preferentially of LPS core type R3, while strains of serogroup O25 were preferentially of LPS core type K-12 or R1. The R1 core type was observed in most O2, O6, O8 and O75 strains. On the contrary, the highly prevalent O1 serogroup showed no preferred association with any LPS core type.

### Virulence gene repertoire-based clustering highlights association of specific LPS core types with specific lineages

We analysed the distribution of LPS core types in a genome-wide, SNP-based phylogenetic analysis of the 269 *E. coli* strains. The genome sequence GCA_001286085, annotated as an *E. albertii* sequence in the GenBank database and identified as such by ClermonTyping, was used as an outgroup. Again, this analysis clearly differentiated the seven major phylogenetic groups described in the *E. coli* species. The STc to which each strain belongs was determined *in silico*.

We further investigated the link between phylogeny, specific LPS core types and the carriage of specific virulence genes by defining the repertoire of virulence genes carried by each strain based on the virulence gene dataset published by Leimbach and colleagues [29]. Considering that they would carry less information, we arbitrarily removed genes whose prevalence was either (i) above 90 % in strains from each phylogroup or (ii) below 30 % in all phylogroups, a subset of 596 genes remained.

**Fig. 4.**
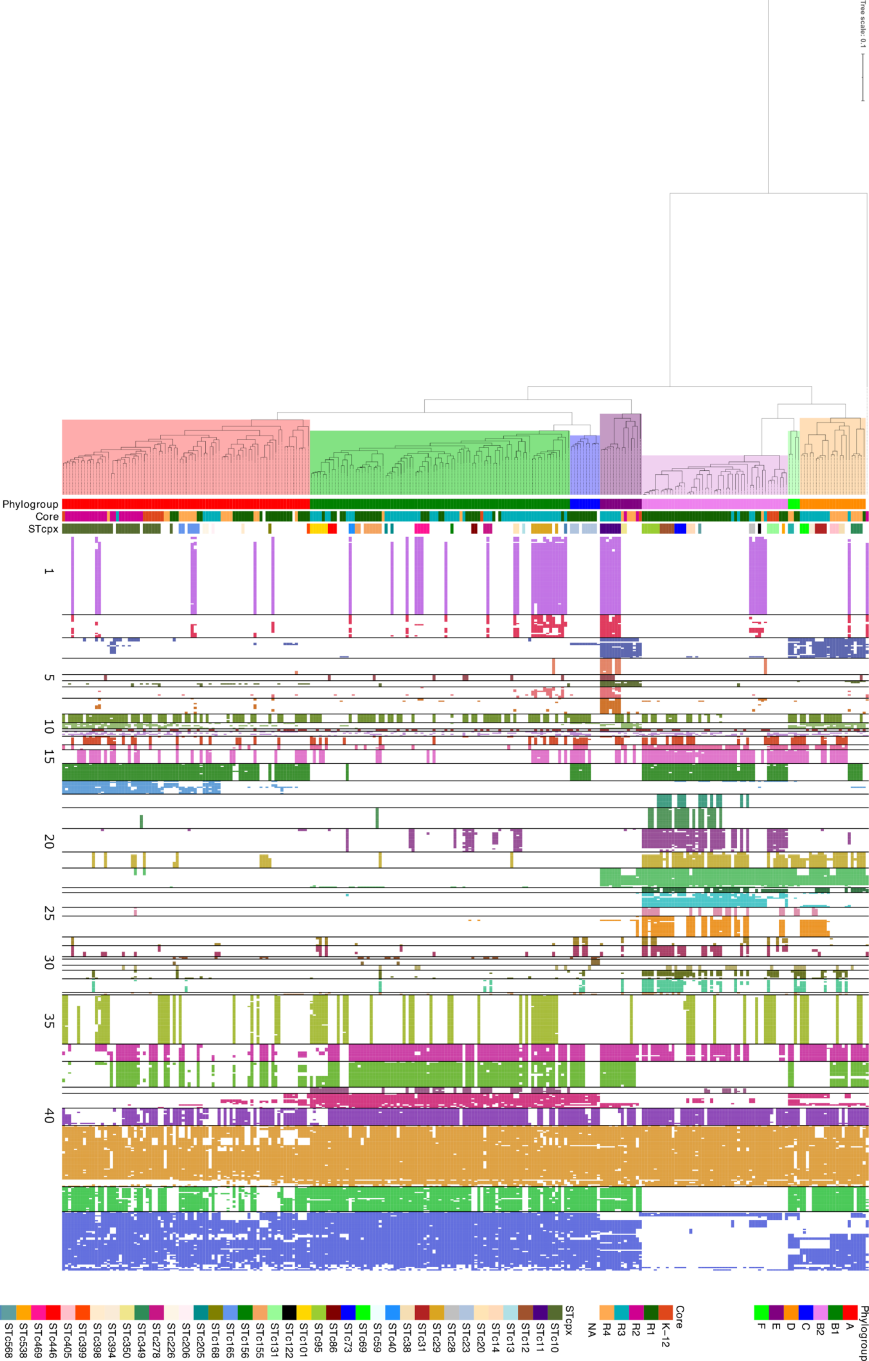
Virulence gene repertoire of strains in relation with phylogenetic clustering. The phylogenetic tree of the 269 *E. coli* genome sequences was determined by kSNP analyses. Presence/absence of virulence genes was based on blastp analysis with a threshold of 80 % identity and 80 % coverage. Presence of a gene is indicated by a coloured cell, the colour depending on the VGG to which the gene belongs. Horizontal lines delineate the different VGGs. The strain of *E. albertii* was included as an outgroup. Graphical representation of the ML tree was performed using the iTOL web server [26]. The branch lengh unit is expressed as the number of SNP between two genomes divided by the total number of SNPs positions in the whole dataset.

**Fig. 5.**
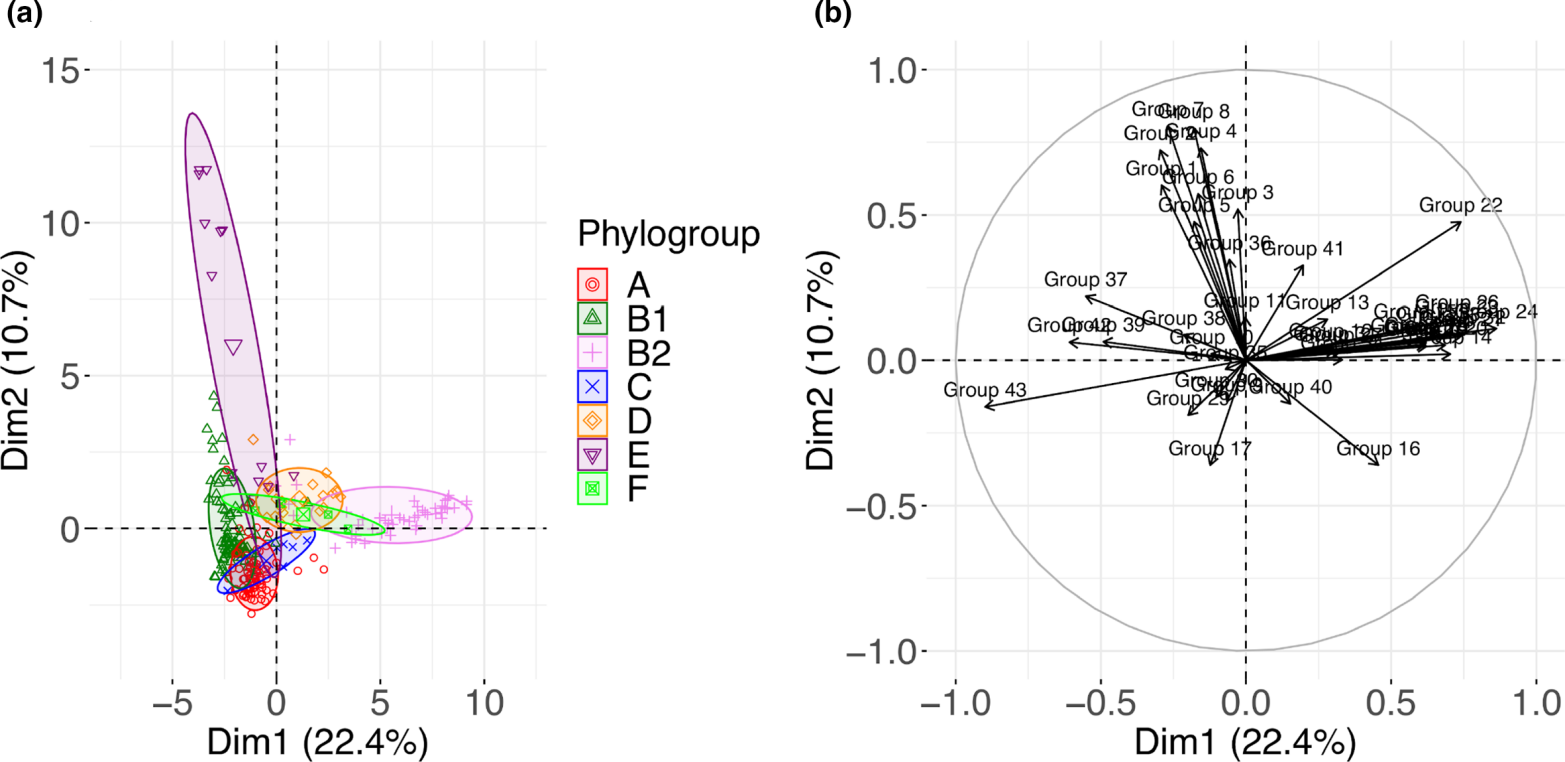
PCA to assess the relationships between phylogroups and enrichment in virulence genes. Enrichment of each strain in the different gene groups was calculated by counting the number of virulence genes from a specific VGG in each strain. PCA was performed on the enrichment scores values using the FactoMineR package. Strains are coloured by phylogroup (**a**) and contribution of the different VGGs is indicated (**b**).

A hierarchical clustering analysis of virulence genes performed on the 596 remaining genes identified 43 VGGs based on their presence/absence patterns (Fig. 4). Relevant features of each VGG are presented in Table 1. The complete list of virulence genes in each VGG is presented in Table S4. The hierarchical clustering of VGGs was overlayed with the phylogenetic distribution of strains, which shows some clearly defined associations of VGG with phylogroups (Fig. 4). To better quantify the association between VGGs, phylogroups and LPS core types, an enrichment score in genes from each VGG was calculated for each strain and analysed by PCA (Fig. 5a, b).

**Table 1.** Relevant features of significant VGGs

Group ID	Relevant features
Group 1	LEE genes and T3SS effectors
Group 2	LEE genes and T3SS effectors
Group 4	T2SS from EDL933 (*etp* cluster)
Group 5	Shiga-toxin genes
Group 6	*fbp* iron-acquisition system
Group 7	T3SS effectors
Group 8	LEE genes and T3SS effectors
Group 9	*fec* iron-uptake system
Group 13	Aerobactin iron-uptake system
Group 14	*sitABCD* iron-uptake system
Group 15	HPI
Group 17	MG1655 putative fimbrial structure
Group 18	*sfa* and *foc* fimbrial clusters
Group 19	Colibactin operon
Group 20	T6SS type i2 from *E. coli* 536
Group 21	*kps* gene cluster
Group 22	*chu* and *fit* iron-uptake systems
Group 24	*auf* fimbrial cluster
Group 25	K1 capsule cluster
Group 26	T6SS type i1 from *E. coli* APEC O1
Group 28	*iro* iron-uptake system
Group 33	P fimbriae gene cluster
Group 35	Lateral flagella
Group 37	T6SS type i2 from *E. coli* ECC-1470 and EDL933
Group 38	*lpf* cluster 1 from *E. coli* ATCC9637
Group 39	*lpf* cluster 2 from *E. coli* ATCC9637
Group 40	*gsp* T2SS from *E. coli* ATCC9637
Group 41	Type 1 fimbriae, flagella, *csgAD* curli, *fhuA* and *cirA* iron-uptake systems
Group 43	*sfm* fimbriae, *gfc* serum resistance cluster

A clear clustering of strains based on phylogroup was observed, in particular for phylogroup B2 strains, which were located on the positive side of the Dim1 axis. Some overlap was observed for A, B1 and C strains, based mostly on the presence of VGGs 9 and 17 (representing the *fec* iron-acquisition system and MG1655 putative fimbriae, respectively), while B1 strains were mostly positive for VGGs 37–39 [type VI secretion systems (T6SSs) and *lpf* gene clusters]. Phylogroup E strains were enriched in VGGs 1–8, which include most type III secretion system (T3SS) structural and effector genes. B2 strains were enriched in VGGs 14–33 (iron-acquisition genes, HPI, colibactin cluster, *kps* genes, K1 capsule cluster, P fimbriae genes) and were mostly devoid of VGGs 1–8, VGGs 37–39 and VGGs 42–43 genes.

**Fig. 6.**
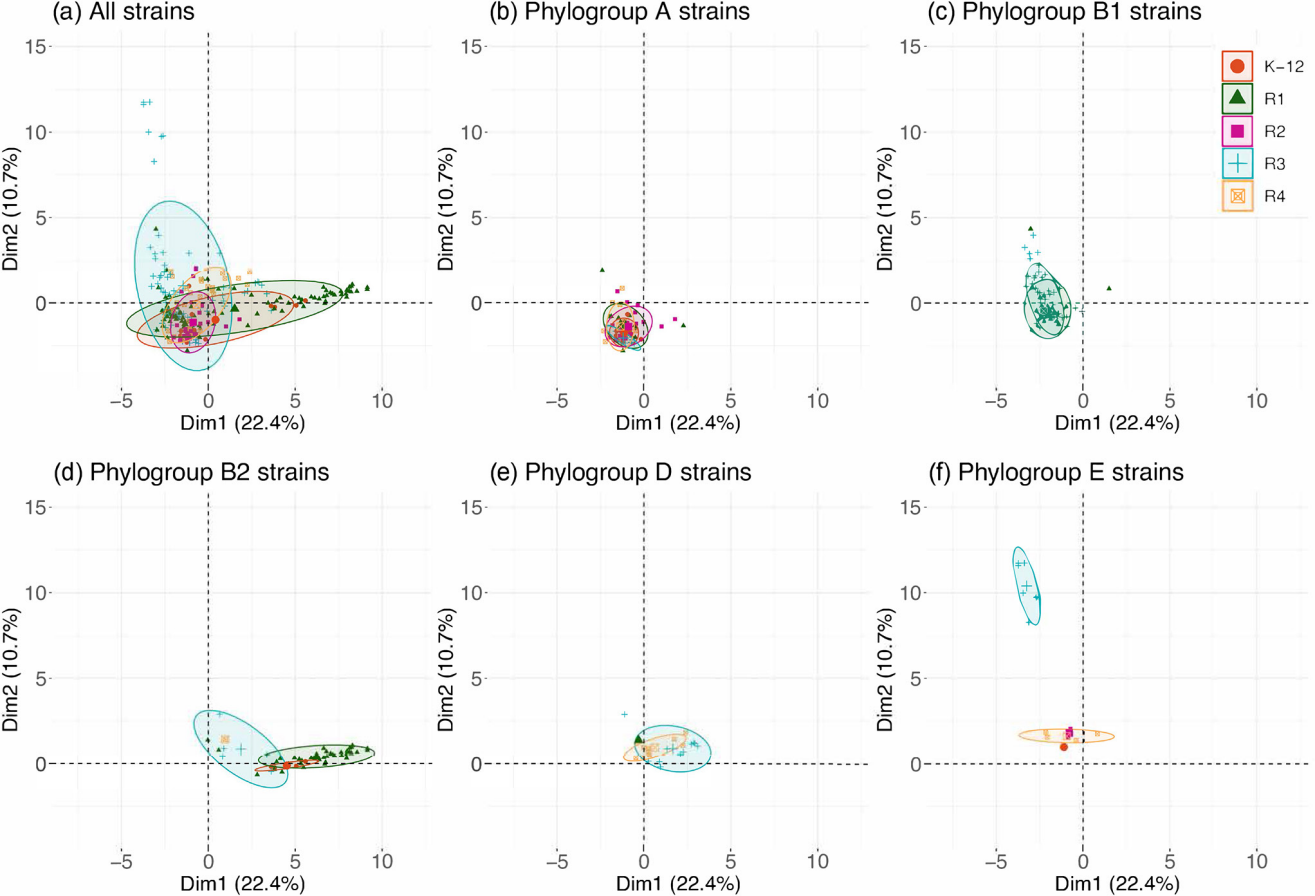
PCA to assess the relationships between phylogroups and LPS core types. Enrichment of each strain in the different gene groups was calculated by counting the number of virulence genes from a specific VGG in each strain. PCA was performed on the enrichment score values using the FactoMineR package. (**a**) Strains belonging to all phylogroups are represented. (**b–f**) Strains belonging to phylogroups A, B1, B2, D and E, respectively, are represented.

We then overlaid the type of core LPS onto the PCA plot to detect potential associations between VGGs and LPS core types. As shown in Fig. 6, no well-defined clusters were distinguished when considering all strains together (Fig. 6a).

When strains for specific phylogroups were analysed separately, some clustering was observed. For example, phylogroup E shows a clear difference in virulence gene content between strains of LPS core type R3 and the others, driven by the presence of VGGs 1–6 in R3 types only (Fig. 6f). More generally, this finding can be extended to all strains carrying LEE (VGG 1): LEE-positive strains are preferentially of the R3 core type compared to LEE-negative strains and this difference is significant (Fisher's exact test; *P*<0.001); out of the 42 strains carrying LEE and distributed in the different phylogroups, 30 have an R3 core type.

**Fig. 7.**
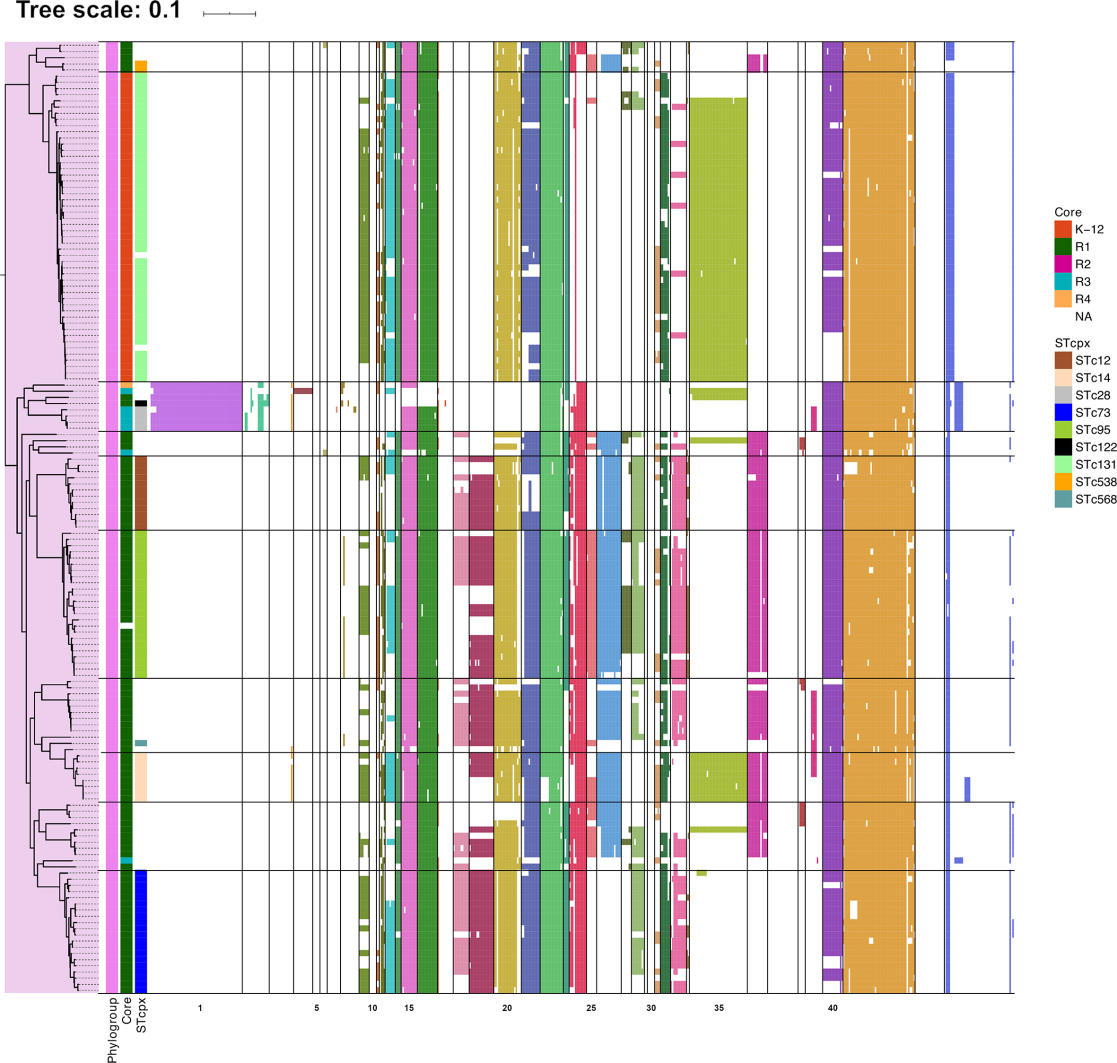
Virulence gene repertoire of strains in relation with phylogenetic clustering of phylogroup B2 strains. All B2 strains from the initial 500 genome dataset are represented. Horizontal lines delineate the different B2 STcs identified. NA: non typable LPS core type. The branch lengh unit is expressed as the number of SNP between two genomes divided by the total number of SNPs positions in the whole dataset.

Another significant clustering was detected with phylogroup B2 strains. Indeed, B2 strain distribution allowed the distinction of three clear clusters, one for each of LPS core types K-12, R1 and R3 (Fig. 6d). Interestingly, B2 strains with a K-12 LPS core all belonged to the STc131 complex. When the 500 strains from our initial dataset were considered, this finding was confirmed (Fig. 7). This cluster of B2 strains with K-12 LPS core types, all belonging to STc131, is enriched in VGGs 9, 13 and 35 containing the *fec* operon, the lateral flagellar genes and the aerobactin operon, respectively (Fig. 7). A group of B2 strains related to the STc28 with a R3 LPS core type are the only ones carrying the VGG 1–2 genes corresponding to the T3SS and some of its effectors. These strains also possess an O-antigen capsule cluster (*etk*, *etp* and *gfcBCDE* genes – found in VGG 43) and are devoid of any of the five T6SS analysed.

Other B2 strains, belonging to STc73, STc95, STc12 and STc14, almost all carried an R1 core type and were enriched in VGGs 18–19, 24–26, 28, 33 and 36 corresponding, among others, to the colibactin operon and several fimbriae gene clusters (Fig. 7).

### Distribution of T6SS and iron-acquisition genes is lineage specific

**Fig. 8.**
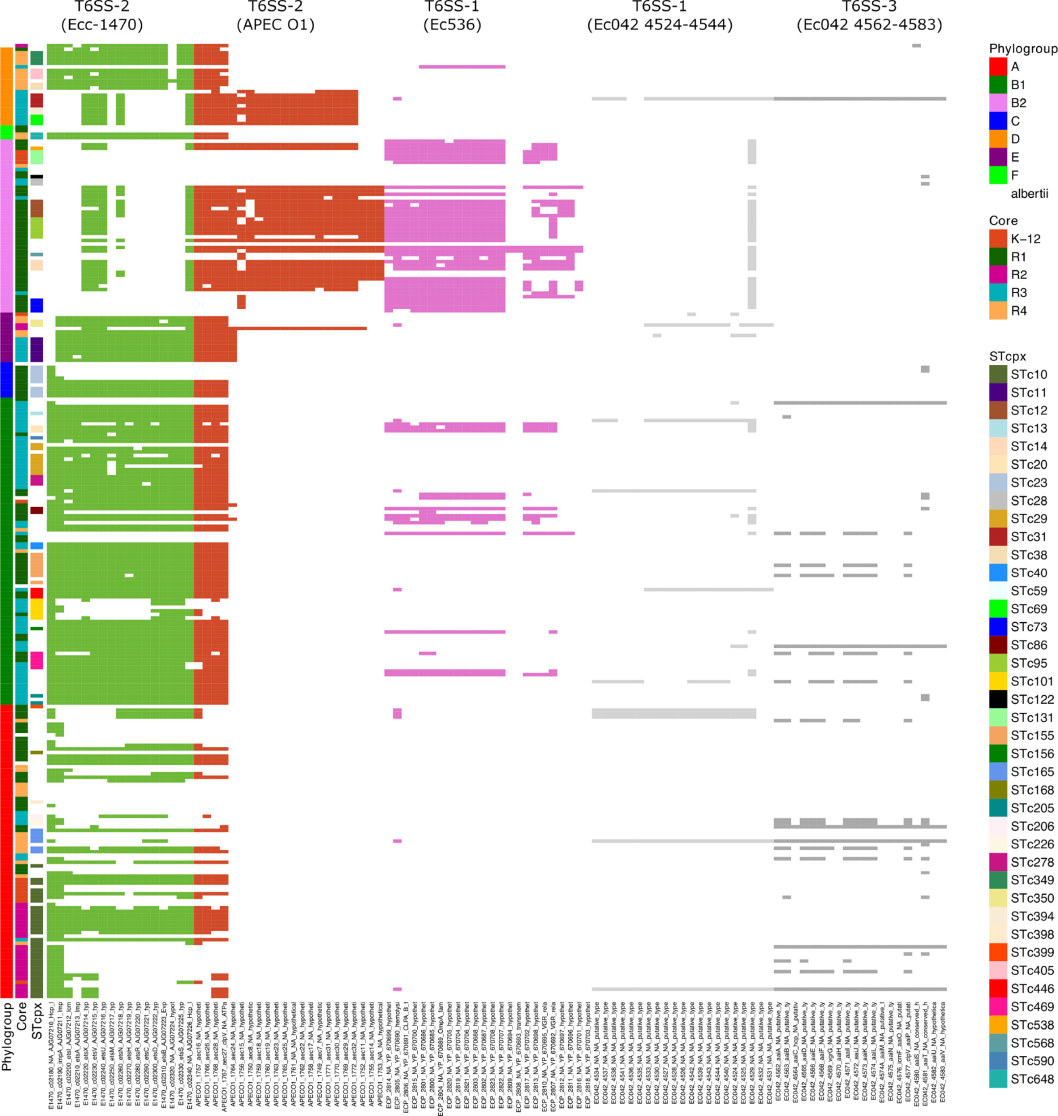
Distribution of T6SSs in the *E. coli* species. Strains are ordered as in Fig. 4. Presence/absence of virulence genes was based on blastp analysis with a threshold of 80 % identity and 80 % coverage. Presence of genes of the five different T6SSs is indicated by a coloured cell, the colour depending on the T6SS to which the gene belongs.

Among the virulence genes analysed, the distribution of T6SSs and iron-acquisition systems highlighted features that, although not directly related to LPS core types, are of particular interest. First, our virulence dataset covered five different T6SS operons, which were not distributed evenly between strains of the different phylogroups (Fig. 8). The T6SS from strain 536, belonging to VGG 20, was mostly present in B2 strains. On the contrary, the T6SS type from strain Ecc-1470 (VGG 37) was absent from B2 strains, while it was present in most E and B1 strains and in some A, D and C strains. The T6SS from strain APEC-O1 (VGG 26) was largely present in B2 strains, with the notable exception of strains from STc131 and STc73. Interestingly, B2 strains from STc28 did not possess any of the five T6SSs. Among D strains, strains possessed either T6SS from APEC-O1 or from ECC-1470, but never both. The two T6SSs from strain 042 were the least frequent (and, thus, are not depicted in Fig. 4 nor listed in Table S4). A closer look at the genetic structure of T6SSs Ecc-1470, APEC-O1 and Ec536 revealed that the first two belong to the T6SS-2 genetic organization while the latter belongs to the T6SS-1 genetic organization, according to the classification of Journet and Cascales [31]. Genomic comparisons with strains lacking T6SS also indicated that the two variants of T6SS-2 (Ecc-1470 and APEC-O1) are both integrated in the same tRNA-Asp, which could explain their mutual exclusion among *E. coli* strains. The T6SS-1 variant Ec536 is, however, inserted in the tRNA-Met allowing the concurrent carriage of T6SS-1 and T6SS-2 variants in *E. coli* strains.

**Fig. 9.**
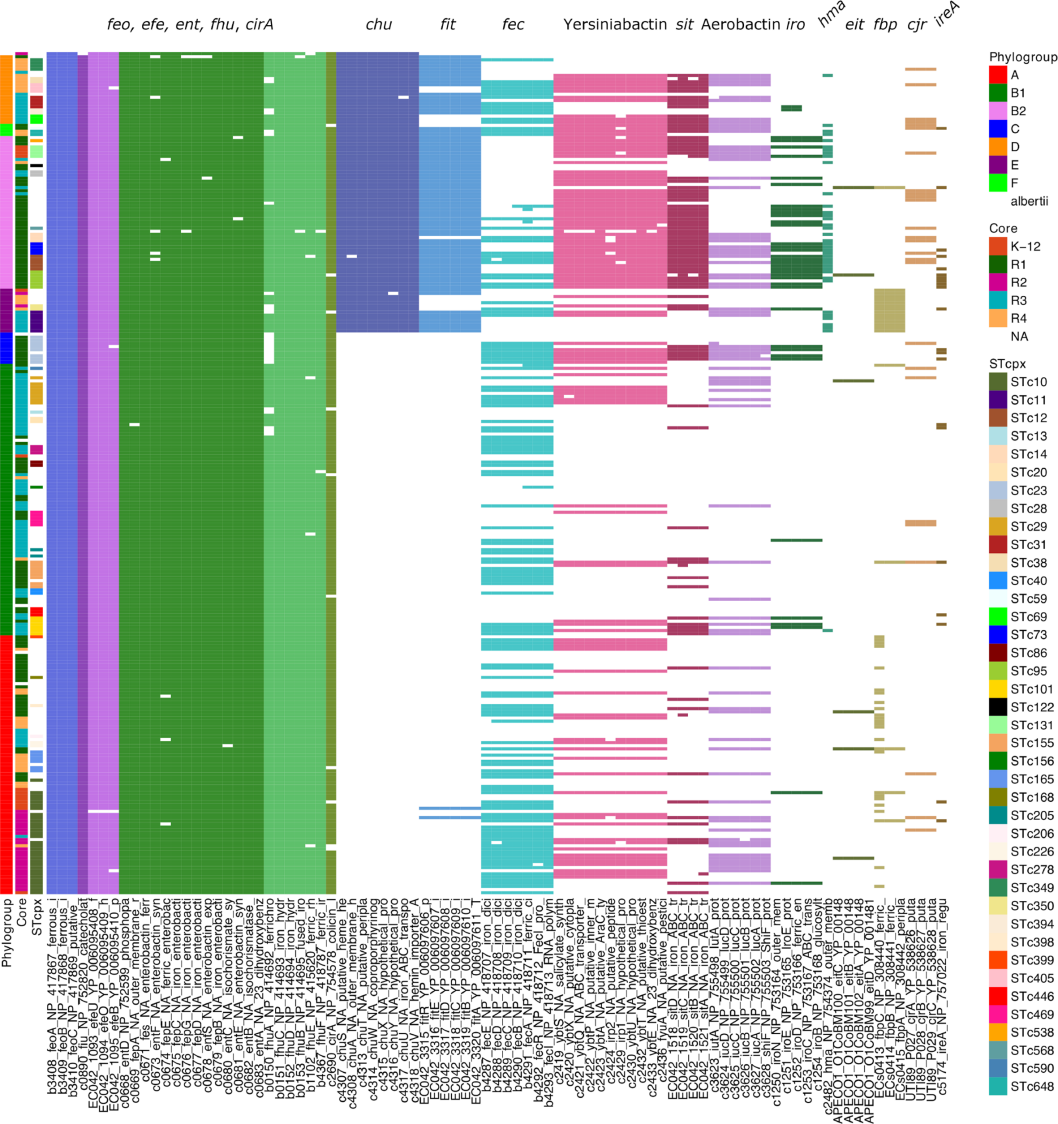
Distribution of iron-acquisition systems in the *E. coli* species. Strains are ordered as in Fig. 4. Presence/absence of all the iron-uptake genes studied in this report was based on blastp analysis with a threshold of 80 % identity and 80 % coverage. NA: non typable LPS core type.

Concerning iron-acquisition systems, our analysis confirmed that some are ubiquitous, such as *fep*, *feo* and *ent* operons, while others are restricted to certain phylogroups (Fig. 9). In particular, phylogroup E strains all possessed the *fbpABC* locus, while they lacked the yersiniabactin, aerobactin, ferric citrate and *sitABCD* operons. Consistent with the Clermont typing scheme, strains from phylogroup A and B1 always lacked the *chu* operon.

## Discussion

The initial purpose of this study was to investigate the distribution of the different LPS core types in the *E. coli* species. After selecting a random set of 500 *E. coli* genomes from GenBank and restricting our dataset to 269 *E. coli* genomes with more than 10 SNP differences, we set out to examine the prevalence of the difference core types, each core type being characterized by the presence of the O-antigen *waaL* specific ligase, as previously described [27].

The high prevalence of the R1 core type we observed had already been described in previous studies analysing avian pathogenic *E. coli* strains or clinical and commensal isolates [17–19]. Another finding consistent with previous reports is the low prevalence of R2 and R4 core types. The high prevalence of R3 core types we observed might have been contributed by an overrepresentation of EHEC/STEC (Shiga-toxin-producing *E. coli *) epidemic strains belonging to phylogroup E, as has been observed previously (see below).

The initial phase analysing the distribution of LPS core types among *E. coli* phylogroups clearly showed a non-random partitioning. As expected, we were able to distinguish seven of the major phylogenetic groups of the *E. coli* species, A, B1, B2, C, D, E and F. This clustering was confirmed by *in silico* phylogroup determination based on the scheme by Clermont *et al.* [28]. An initial observation was an almost even representation of all five core types among phylogroup A strains, while other phylogroups were enriched in some specific core types, in particular E and B1 strains in R3 core types and B2 strains in R1 types. This high diversity observed in phylogroup A strains parallels the high diversity of gene families and large pan-genome of phylogroup A strains recently described by Touchon *et al.* [32]. We believe this uneven distribution of LPS core types in the different phylogroups is not linked to a limited number of strains in some of the phylogroups, but is rather indicative of a true association of certain lineages with specific LPS core types, as observed for STc131, STc28 or LEE-positive strains (see below). Although phylogroup C and F strains seemed underrepresented in our dataset, this distribution actually reflects the genetic diversity of this species revealed so far [32]. Furthermore, an additional search on the EnteroBase database retrieved only six new non-clonal strains of each of the two phylogroups: all 6 C and 5 out of 6 F strains carried an R1 core. The remaining F strain carried an R4 LPS core type. Such a distribution confirms our initial observations.

LPS core types were also unevenly distributed among serogroups. While only the most prevalent serogroups were analysed, we found that only the O1 O-antigen could be associated with any of the five LPS core types. Conversely, the O157, O73 or O103 O-antigens were preferentially attached to the R3 core type.

A more detailed analysis showed that, within each phylogroup, phylogenetic clusters of strains were observed with specific LPS core types. This is notably true for specific clonal complexes of phylogroup B2 strains where the STc131 complex is associated with K-12 LPS core types and STc28 with R3 core types, while most other B2 strains are of the R1 type. Phylogroup D strains also show cluster correlation with specific LPS core types, with R4 and R3 strains belonging to distinct clades in the D phylogroup. Clustering was also obvious in phylogroup E strains with two phylogenetic clades encompassing either strains with an R3 core type and or with R4 types.

One question that justified the present study was whether we could identify a link between specific LPS core types and virulence properties or adaptation to specific environmental niches. When virulence gene repertoires were analysed, we could identify co-presence patterns by distinguishing different VGGs. Based on virulence gene repertoire data, clear clustering based on phylogroup was observed confirming that strains belonging to the same phylogenetic group are enriched in some virulence gene sets, consistent with previous studies [30, 32].

Concerning the link between the virulence gene repertoires and the LPS core type, a significant association of the R3 core type with LEE (VGG 1) positive strains was observed, although LEE-positive strains with other LPS core types were identified. One hypothesis that could explain such a bias would be that acquisition of the LEE locus preferentially required an R3 core. Indeed, acquisition of the LEE locus through phage transfer has been suggested and one could suggest that such transducing phages could require an R3 core as a receptor [33, 34]. Curiously, none of LEE-positive phylogroup A strains possessed an R3 core, but rather possessed R1, R2 or R4 core types in roughly equal proportions. Whether this represents distinct acquisition events is an open issue.

Despite this lack of recognizable association between virulence gene repertoire and LPS core types, we further deciphered the diversity of B2 strains and possible relationships between their virulence gene repertoire, LPS core types and serogroup. Another finding of the present work is the specific association of the core LPS K12 with STc131 within phylogroup B2, whatever the serogroup (O1, O25, or O16). STc131 clonal complex is one of the four major ExPEC clones that have been identified in various continents and show frequent resistance to multiple drugs [1]. The reason for this strong association between STc131 strains and K-12 core types would deserve further investigation to understand whether the K-12 core type of STc131 strains could contribute to the specific properties of these strains, in particular fitness or antibiotic resistance through altered outer-membrane permeability, for example, or to the emergence of these strains.

In addition to the association of STc131 with K-12 core type, our results show unequal distribution of all five core types observed in the different phylogroups. The origin of this uneven distribution is unclear. It is well recognized that *E. coli* has an ‘open’ genome and that horizontal gene exchange is common, even within phylogroups; hence, it is not surprising that LPS core types may not be ‘fixed’ in individual phylogenetic backgrounds [35]. Fig. S1 clearly shows a ‘core genome’ level G+C ratio of approximately 51 mol% at the start (*waaDFC*) and end (*waaA*) of the *waa* loci. A slight reduction at *waaQGP* is observed followed by a marked reduction to approximately 17 % in the central *variable* loci. This would suggest that the diversity of the *waa* locus originated from horizontal gene transfer (HGT). Yet, a survey of annotations within, adjacent and more distant to *waa* loci did not identify anything that resembles phage, IS or Tn remnants, supporting an acquisition of the variable region through transformation or transduction followed by integration through homologous recombination, as described for the transfer of large genome fragments [36]. HGT though homologous recombination of the central region is also the most likely explanation for the LPS core types exchange between *E. coli* strains. Finally, no homologue of this central region was detected in any bacterial genome available in GenBank at the time of the study, other than in *Escherichia* and *Shigella* species. The origin(s) of the variable region, therefore, remains unknown.

The consequences for some of the above-mentioned associations remain to be decrypted, although it seems relevant to speculate whether specific LPS core types could be associated with ecological niches or other selection processes. Different core structures may, for instance, provide a better ability to interact with different structures such as phage tails or immune cell receptors. Although mainly composed of the hexoses glucose and galactose, the presence of glucosamine and *N*-acetylglucosamine in R3 and R2 outer core regions, respectively, could conceivably affect host interactions in a similar manner to that observed for K-12 core types (which possess a heptose moiety), which show distinct interactions with DC-SIGN [15, 16], for example. The extent to which different core LPSs may confer different properties to the outer membrane of bacteria is an important area for further exploration. For instance, parts of the LPS molecules have been identified as receptors for P1 phage [37]. Different LPS core types, thus, could allow bacteria to escape the binding of these bacteriophages or, conversely, permit phage binding and introduction of phage-specified genome content. When one considers the strong selective pressure imposed by bacteriophages on bacterial evolution, possessing a specific LPS core type could be a potential selective advantage in an environment loaded with bacteriophages not binding this core type. The core type could also influence how bacteria are recognized by host immune cells, as was shown for K-12 core types interacting with the DC-SIGN receptor at the surface of dendritic cells [15, 16].

A bystander observation of our study was that T6SS distribution varied between strains of different phylogroups and lineages. Based on their G+C content, proximity to tRNA, Rhs elements or putative transposase, it is considered that T6SS have been acquired by HGT [31, 38]. Of particular interest is the mutual exclusion of the two T6SS-2 (Ecc-1470 and APEC-O1) because of their shared insertion site. T6SSs have recently been shown to contribute to the adaptation of strains to their niche, such as the intestinal microbiota. T6SS, thus, could be a major player for adaptation to different niches, each being colonized by different microbiota [39]. To that end, it is interesting to note that, based on strains isolated from different sources in Australia, Touchon *et al.* showed strong associations between the phylogenetic structure of populations and the natural habitats of strains [32]. The presence/absence pattern of T6SS-2 (Ecc-1470 or APEC O1) and TSS-1 (Ec536) within phylogroup B2 is of particular interest, as other authors have argued that the virulence of B2 strains might be a by-product of increased fitness [40]. Considering the role of the T6SSs as ‘effective weapons for bacterial competitiveness’ [41], it is very likely that the presence of two T6SSs have indeed contributed to the increased fitness of B2 strains. In addition, the presence of only T6SS-1 (Ec536) in major ExPEC lineages such as STc73 and STc131 suggests that if the T6SS-2 (APEC O1) indeed contributed to fitness of these strains, T6SS-1 (Ec536) might be of particular importance. Contrastingly, the absence of any T6SS in STc28 strains, also characterized as LEE-positive B2 strains with an R3 core, would suggest these strains might be less able to compete or have developed other means of competition/fitness. Hence, the interplay between T6SS, phylogenetic group and adaptation to a specific niche will be worth investigating in more detail.

Likewise, we observed a clear pattern of iron-acquisition genes within several phylogenetic groups. Such observations were previously made for EHEC and UPEC (uropathogenic *E. coli *) strains in which the *fec* iron-transport system was absent or for B2 strains in which the *chuA* gene is absent and serves as a base for the Clermont typing scheme [42].

Altogether, this report has highlighted the diversity of LPS core types in the *E. coli* species with specific associations between LPS core types and strains of certain phylogroups. Based on the clustering of some LPS types with specific groupings, such as K-12 core types and STc131 strains, our results support the relevance of analyses of the biological properties of LPS molecules belonging to different LPS core types. This reveals the need for further genotype–phenotype investigations targeting surface structures such as the LPS core and ability to occupy distinct niches.

## Supplementary Data

Supplementary material 1

Supplementary material 2

Supplementary material 3

Supplementary material 4

Supplementary material 5
