# Supplementary material for: Lipopolysaccharide core type diversity in the Escherichia coli species in association with phylogeny, virulence gene repertoire and distribution of type VI secretion systems
Source: Microb Genom. 2021 Sep 29;7(9):000652. doi: 10.1099/mgen.0.000652 (PMC8715443; doi:10.1099/mgen.0.000652)

Supplementary Figure 1 - - Leclercq et al., Lipopolysaccharide core type diversity in the Escherichia coli species in association with phylogeny, virulence gene repertoire and distribution of type VI secretion systems

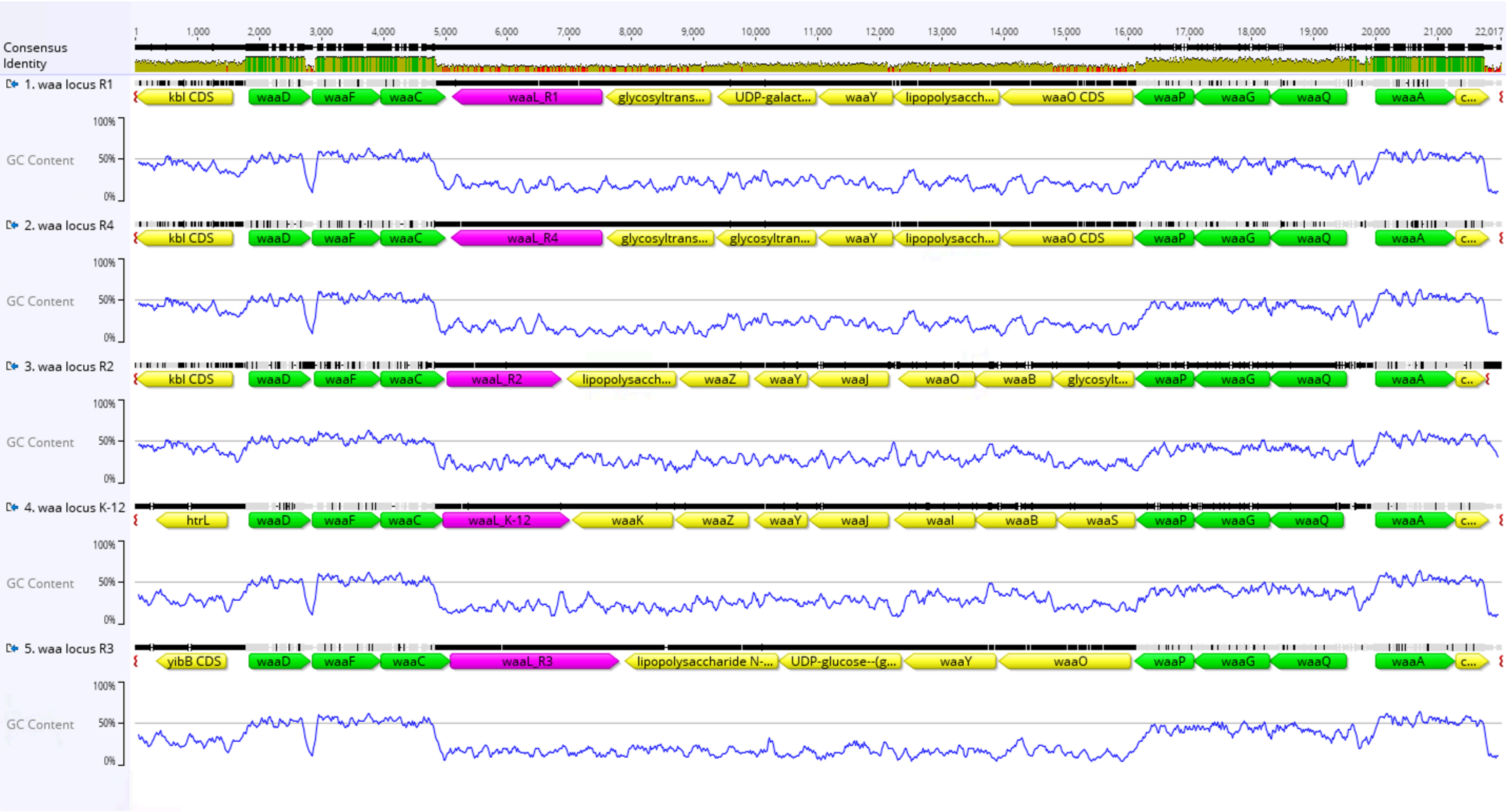

Supplement: Supplementary material 1 [file mgen-7-0652-s001.pdf]
